# Pseudo-Meigs’ syndrome due to ovarian metastases from colon cancer: a case report and review of the literature

**DOI:** 10.1186/s40792-016-0209-7

**Published:** 2016-10-12

**Authors:** Atsushi Yamamoto, Yoshiaki Miyasaka, Kazushige Furuya, Hideki Watanabe, Masahiro Maruyama, Haruka Nakada, Atsushi Takano, Masao Hada, Hiroshi Nakagomi, Masao Omata, Toshio Oyama

**Affiliations:** 1 Department of Surgery, Yamanashi Prefectural Central Hospital, 1-1-1 Fujimi, Kofu, Yamanashi 400-8506 Japan; 2 Department Internal Medicine, Yamanashi Prefectural Central Hospital, 1-1-1 Fujimi, Kofu, Yamanashi 400-8506 Japan; 3 Department Pathology, Yamanashi Prefectural Central Hospital, 1-1-1 Fujimi, Kofu, Yamanashi 400-8506 Japan

**Keywords:** Pseudo-Meigs’ syndrome, Ascending colon cancer, Ovarian metastases

## Abstract

We herein experienced a case with pseudo-Meigs’ syndrome that developed both synchronous and metachronous metastases to the ovary from ascending colon cancer. A 57-year-old female visited a hospital for a 2-month history of abdominal distension and voiding difficulty. Massive pleural effusion on the right side and a small amount of left-sided pleural effusion were detected on CT. She underwent emergent laparotomy due to the severe symptom of abdominal distention. The tumor originated from the left ovary, and left-sided oophorectomy was performed.

The histologic finding was moderately differentiated adenocarcinoma suggesting metastatic carcinoma from the colon. Left thoracic effusion disappeared at 3 days after the removal of the ovarian tumor. Subsequently, colon carcinoma of the cecum was detected by colonoscopy. The patient underwent second laparotomy of right colectomy and lymph node dissection. However, 6 months after the operation, pleural effusion on the right side re-developed again, and the serum levels of CEA and CA125 were elevated at 105 ng/ml and 125 U/ml, respectively. CT again revealed a large ovarian tumor. She subsequently underwent third laparotomy of right-sided oophorectomy and hysterectomy. Pleural effusion and ascites disappeared in a few days after the operation.

The patient developed both synchronous and metachronous ovarian metastases and achieved a 7-year disease-free survival after the operation. The pathogenesis of pseudo-Meigs’ syndrome should be distinguished from carcinomatous peritonitis and/or pleuritis of malignant disease.

## Background

Meigs and Cass first reported cases that developed non-malignant ascites and/or pleural effusion in association with benign ovarian tumors such as fibroma, thecoma, and Brenner tumor [1]. These effusions disappeared after the removal of the tumors. Thereafter, this symptom was associated with benign ovarian tumors and referred to as Meigs’ syndrome. The same clinical pictures due to a malignant ovarian or pelvic tumor have been referred to as pseudo-Meigs’ syndrome [2, 3].

The etiology of Meigs’ and pseudo-Meigs’ syndrome is not fully elucidated, and no large case series studies are currently available. We herein report a case with pseudo-Meigs’ syndrome that developed both synchronous and metachronous metastases to the ovary from ascending colon cancer and review of the pertinent literature.

## Case presentation

A 57-year-old female visited a hospital with a 2-month history of abdominal distension and voiding difficulty. Because of a large pelvic tumor detected on CT, she was referred to the Department of Gynecology at our hospital.

**Fig. 1:**
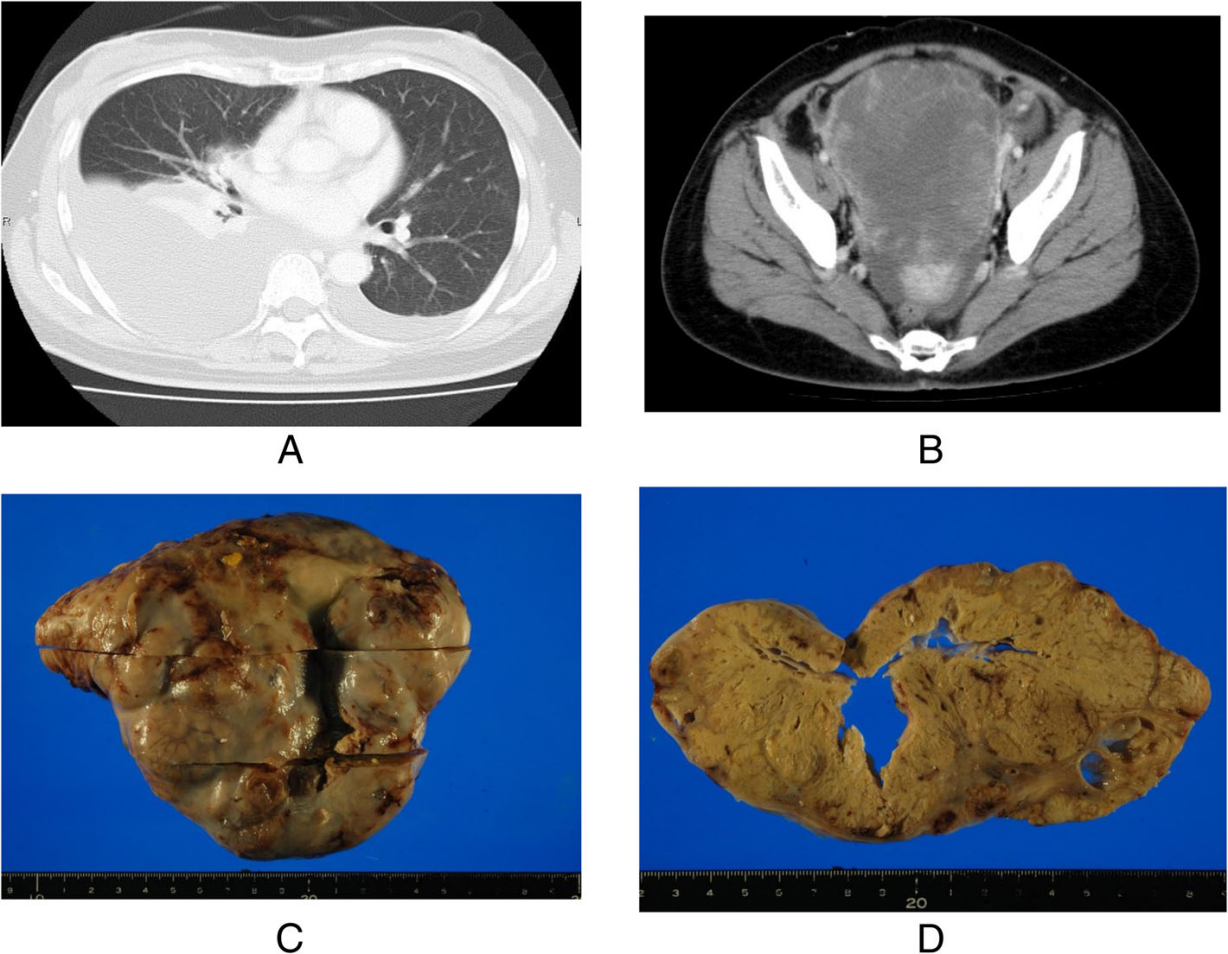
**a** Chest CT revealed massive pleural effusion at the right side and a small amount of left-side pleural effusion. **b** Abdominal CT indicated a large pelvic tumor. **c** The tumor of the left-side ovary measured 17 × 14 cm and contained necrotic tissues (**d**)

**Fig. 2:**
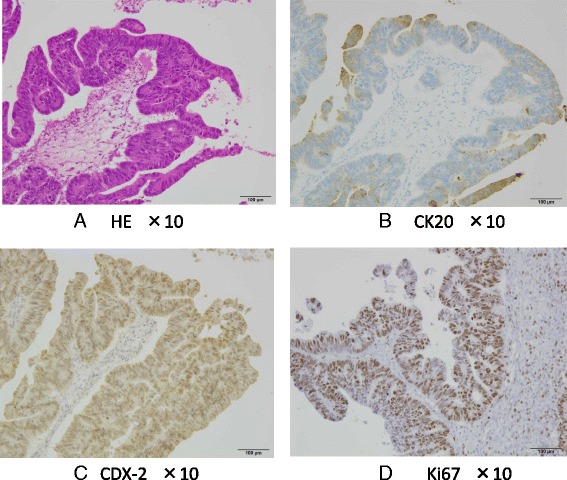
**a** Histologic finding was moderately differentiated adenocarcinoma and CK20 (+) (**b**) and CDX (+) (**c**) with immunohistochemistry, suggested metastatic carcinoma from the colon. The Ki67 labeling index of the tumor was very high (>80 %) (**d**)

Massive pleural effusion on the right side and a small amount of left-sided pleural effusion were detected on CT (Fig. 1a). Furthermore, CT revealed a large pelvic tumor measuring 13 cm in diameter (Fig. 1b) and ascites. The cytology of pleural effusion showed no malignant cells. The serum tumor marker levels of CEA, CA19-9, and CA125 were 28.7 ng/ml, 60.6 U/ml, and 302 U/ml, respectively. She subsequently underwent emergent laparotomy due to the severe symptom of abdominal distention. The tumor originated from the left ovary, and left-sided oophorectomy was performed. Cytology of ascites showed no malignant cells, and the tumor measured 17 × 14 cm (Fig. 1c) and contained necrotic tissue (Fig. 1d). The histologic finding was tubular adenocarcinoma (Fig. 2a), and CK20 and CDX-2 were positive by immunohistochemistry (Fig. 2b c), suggesting metastatic carcinoma from the colon. The Ki67 labeling index of the tumor was also very high (>80 %) (Fig. 2d).

**Fig. 3:**
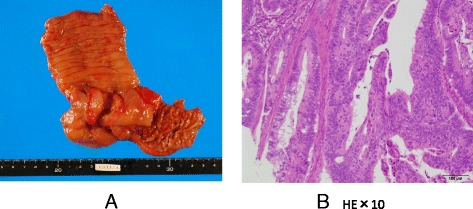
**a** Type 2 colon cancer measuring 4 × 4.5 cm was seen. **b** Histologic finding indicated mod. ss, ly (+), v (2+), *n* + (3/33)

**Fig. 4:**
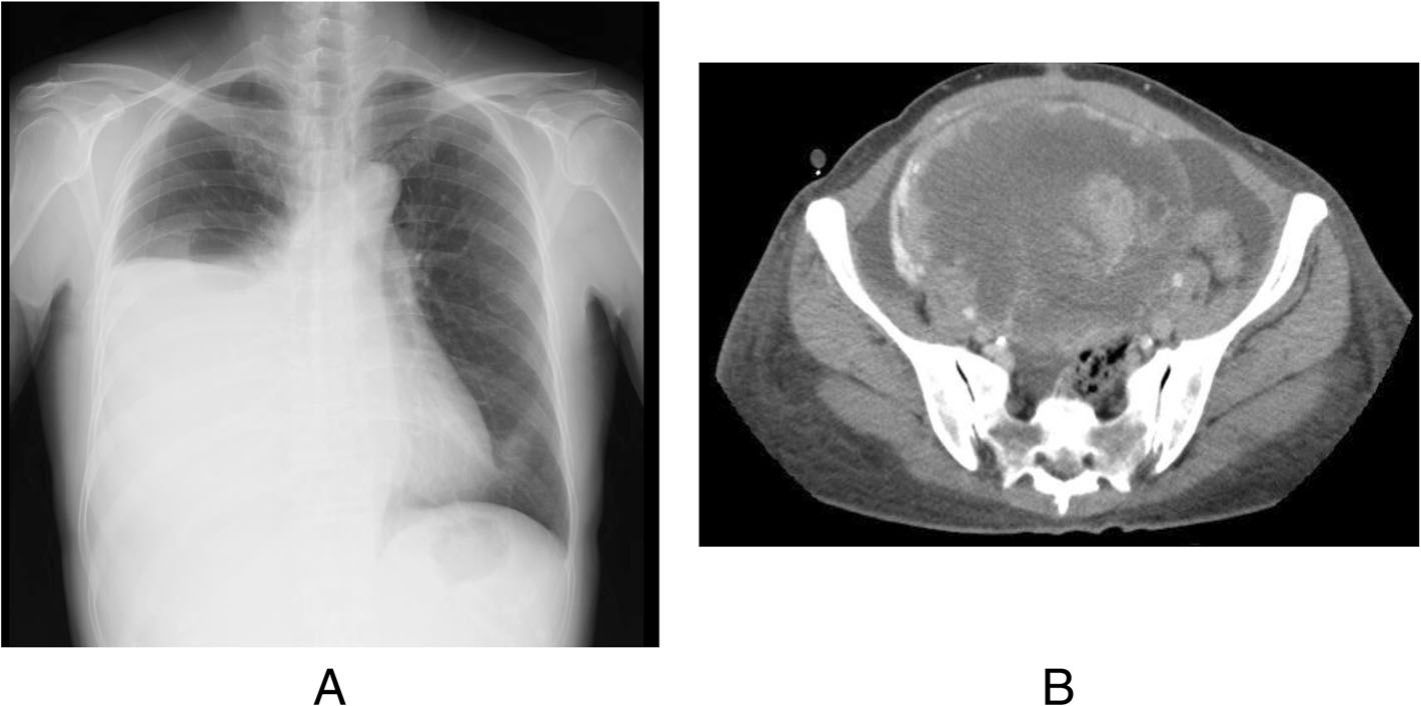
**a** Six months after the operation, pleural effusion of the right side has developed again. **b** CT revealed huge ovarian tumor again

Left thoracic effusion disappeared at 3 days after the removal of the ovarian tumor. Subsequently, we examined the whole body to find the primary tumor site, then colon carcinoma of the cecum was detected by colonoscopy. The patient underwent right colectomy and lymph node dissection again. The operative finding indicated a normal right ovary. The pathologic finding indicated type 2 colon cancer measuring 4 × 4.5 cm (Fig. 3a), moderately differentiated adenocarcinoma with subserosal invasion, vascular permeation, and lymph node metastases (3/33) (Fig. 3b). She was treated with oral uracil/tegafur and leucovorin. However, 6 months after the operation, pleural effusion of the right side again developed (Fig. 4a), and the serum levels of CEA and CA125 were elevated at 105 ng/ml and 125 U/ml, respectively. CT again revealed a large ovarian tumor (Fig. 4b). The patient subsequently underwent right-sided oophorectomy and hysterectomy at 9 months after the left-sided oophorectomy. The tumor on the right-sided ovary measured 21 × 17 cm and was histologically confirmed to be metastatic colon cancer, the same as the previous tumor from the left ovary.

Pleural effusion and ascites disappeared in a few days after the operation. She received 9 cycles of FOLFOX-4 chemotherapy (oxaliplatin 80 mg/m^2^ div at day 1, 5-Fu 600 mg/m^2^, and levofolinate 100 mg/m^2^ div at days 1 and 2) and has remained alive for 7 years with no recurrence.

### Discussion and review of the literature

Pseudo-Meigs’ syndrome is defined as a condition with ascites and pleural effusion that is improved after the removal of an ovarian or pelvic tumor including both benign and malignant ovarian tumors. The primary sites of malignant disease can vary and include an ovarian malignant tumor [4], leiomyoma [5], gastric cancer [6], breast cancer [7, 8], and colorectal cancer [9].

Although the etiology of development of ascites and pleural effusion was not elucidated well, the following explanation has been proposed. Chronic transudation of interstitial edema of a large tumor may cause the persistent retention of ascites [10]. Alternatively, the stromal edema and transudation was caused by an imbalance between arterial and venous blood flow of a large tumor [11]. Furthermore, pleural effusion develops through trans-diaphragmatic lymphatic vessels [12]. Additionally, a correlation with VEGF has been recently suggested [13].

According to a search using the PubMed and Japanese MEDLINE databases (during the period of 1963–2015), 28 cases were reported cases (7 reports published in English and 21 reports published in Japanese with English abstracts). We reviewed the clinical data of the 29 cases, including the present case (Table 1).

**Table 1:** Review of literatures of pseudo-Meigs’ syndrome due to ovarian metastases from colon cancer

Number	Author	Year (ref)	Age (years)	Colon cancer		Onset S or M	Effusions		Serum CA125 (U/ml)	Ovarian tumor			Prognosis (months)
				Primary site	Pathology		Ascites	Pleural		Site R or L	Size (cm) R/L	Surgical procedures	
1	Ryan RJ	1972 [9]	38	T	nd	M	No	Right	nd	R	15 × 20	BSO	Alive (120)
2	Tanimura A	1990 [14]	40	D	Well	S	Yes	Bilateral	1400	R/L	nd	BSO	nd
3	Matsuzaki M	1992 [15]	39	R	mod	S	Yes	Bilateral	nd	R	24 × 22	RO	Alive (12)
4	Furumoto T	1993 [16]	75	R	mod	S	Yes	Right	422	R/L	nd/21 × 12	BSO	Alive (12)
5	Nagakura S	2000 [17]	53	S	Well	S	Yes	Right	nd	R/L	18 × 18/12 × 12	BSO ATH	Alive (52)
6	Koide A	2002 [18]	52	S	Well	S	Yes	Bilateral	902	L	30 × 25	LO	Alive (10)
7	Inokuma S	2002 [19]	42	RS	mod	S	Yes	Right	395	L	16 × 9	BSO ATH	Alive (42)
8	Ohsawa T	2003 [12]	41	S	mod	S	Yes	Bilateral	835	R/L	16 × 12/15 × 13	BSO	Died (8)
9	Hatada T	2003 [20]	52	S	Well	S	Yes	Bilateral	902	L	30 × 25	BSO ATH	Alive (19)
10	Kobayashi Y	2003 [21]	48	A	Well	S	Yes	Bilateral	207	R/L	14 × 7/15 × 11	BSO ATH	Alive (19)
11	Feldman ED	2004 [22]	49	C	nd	M	No	Left	557	R	16	BSO	Alive (6)
12	Shundo Y	2004 [23]	43	S	Well	S	Yes	Right	81	R/L	12 × 10/3 × 2	BSO ATH	Died (24)
13	Motoyama K	2005 [24]	48	A	mod	S	Yes	Right	312	R/L	4 × 7/21 × 19	BSO ATH	Alive (12)
14	Sobajima J	2007 [25]	54	S	mod	S	Yes	Right	787	R/L	18 × 12/nd	BSO	Died (12)
15	Ohsawa T	2007 [26]	32	S	Well	S	Yes	Bilateral	595	R/L	17 × 13/3.5 × 2	BSO ATH	Alive (24)
16	Rubinstein Y	2009 [27]	61	C	nd	S	Yes	Bilateral	954	R/L	nd/17	BSO	nd
17	Ikeda Y	2009 [28]	53	S	mod	S	Yes	Bilateral	618	R/L	21 × 15/30 × 17	BSO	Alive (8)
18	Miki T	2010 [29]	54	D	mod	S	Yes	Bilateral	327	R	18	BSO	Alive (14)
19	Murakami H	2010 [30]	45	S	por	M	Yes	Right	167	R/L	4 × 2/16 × 12	BSO	Alive (6)
20	Murakami H	2010 [31]	53	R	Well	M	Yes	Right	705	R	nd	RO	Died (4)
21	Ishii M	2010 [32]	58	S	Well	S	Yes	Left	nd	L	21 × 20	BSO	Alive (12)
22	Okuchi Y	2010 [13]	43	R	mod	M	Yes	Right	515	L	11 × 8	BSO	Died (12)
23	Komatsu H	2011 [33]	49	A	mod	S	Yes	Right	67	R/L	15 × 12/10 × 8	BSO ATH	Alive (25)
24	Maeda H	2011 [34]	58	S	Well	S	Yes	Right	921	R/L	15/nd	BSO	Alive (10)
25	Iwagami Y	2011 [35]	36	S	mod	M	Yes	Right	427	L	21 × 17	BSO ATH	Alive (10)
26	Saito H	2012 [36]	44	S	Well	S	Yes	Bilateral	576	R	22 × 22	BSO	nd
27	Iinuma A	2014 [37]	55	A	Well	S	Yes	right	912	L	19 × 20	LO	Alive (10)
28	Yachi T	2015 [38]	65	A	Well	M	Yes	Bilateral	669	R	13 × 10	RO	Alive (27)
29	Present case	na	57	A	Well	S, M	Yes	Bilateral	302	R/L	17 × 14/21 × 17	LO RO	Alive (89)

*C* cecum, *A* ascending, *T* transverse, *D* descending, *S* sigmoid, *RS* recto, sigmoid, *R* rectum, *S* synchronous, *M* metachronous, *BSO* bilateral salpingo-oophorectomy, *ATH* abdominal total hysterectomy, *LO* left oophorectomy, *RO* right oophorectomy, *na* not applicable, *nd* not documented

The mean age was 49 ± 9 years (range 32–65 years), and the primary site of colorectal cancers included the cecum 2, ascending colon 6, transversus colon 1, descending colon 2, sigmoid colon 13, and rectum 4 patients. The histologic type of colon cancers was well to moderately differentiated adenocarcinoma, except one case which was reported to be poorly differentiated adenocarcinoma.

The onset of pseudo-Meigs’ syndrome was 75 % (21/28) synchronous and 25 % (7/28) metachronous, while a case that developed both synchronous and metachronous, similar to the present case, was not reported.

Nearly all cases had ascites except two cases that developed only pleural effusion. Additionally, all cases had pleural effusions, unilaterally in 16 cases (55 %) (14 on the right side, 2 on the left side) and bilaterally in 13 cases (45 %). The serum level of CA125 was elevated at 557 ± 323 U/ml (range 67–1400 U/ml) in nearly all cases.

Concerning metastatic ovarian tumors, the mean tumor size measured 18 ± 5 cm (range −30 cm), and 15 of 29 cases (52 %) had metastatic ovarian carcinoma on both sides, while no laterality was found in unilateral cases. According to these results, bilateral salpingo-oophorectomy (BSO) should be performed in cases diagnosed with pseudo-Meigs’ syndrome. The present case, which demonstrated repeated pseudo-Meigs’ syndrome, also emphasizes the necessity of BSO.

The primary clinical challenge for pseudo-Meigs’ syndrome is the misdiagnosis with carcinomatous peritonitis or pleuritis. The condition of patients with pseudo-Meigs’ syndrome is often confused with terminal stage malignant disease, for which surgical treatment is not indicated except for a palliative surgery. In the previously reported cases, pleural effusions and ascites rapidly recovered after the removal of the ovarian tumors. Moreover, the patients achieved long survivals (Table 1), especially the present patient who achieved a 7-year disease-free survival. According to these findings, the pathophysiology of pseudo-Meigs’ syndrome must be distinguished from carcinomatous peritonitis and/or pleuritis of malignant disease. If repeated cytology for effusions is negative for malignant cells, we might have a chance to achieve curative operation.

## Conclusions

We herein reported a case with pseudo-Meigs’ syndrome due to ovarian metastases from colon cancer. The patient developed both synchronous and metachronous ovarian metastases and achieved 7-year disease-free survival after the operation. The pathophysiology of pseudo-Meigs’ syndrome must be distinguished from carcinomatous peritonitis and/or pleuritis of malignant disease.

## Consent

Written informed consent was obtained from the patient for publication of this case and any accompanying images. A copy of the written consent is available for review by the Editorial-in-Chief of this journal.
